# Development of an Optimized in Planta Transformation System in Sugarcane and Its Application on *Sh4CL13* in Chlorogenic Acid Biosynthesis

**DOI:** 10.3390/plants15162489

**Published:** 2026-08-17

**Authors:** Yue-Han Zhao, Lin Li, Ya-Li Wu, Hai-Tao Zhao, Hua-Ying Fu, San-Ji Gao, Jin-Da Wang

**Affiliations:** National Engineering Research Center of Sugarcane, Key Laboratory of Sugarcane Biology and Genetic Breeding, Fujian Agriculture and Forestry University, Fuzhou 350002, China

**Keywords:** Sugarcane, in planta transformation, *Agrobacterium*-mediated transformation, *Sh4CL13*, chlorogenic acid, insect resistance

## Abstract

Sugarcane (*Saccharum* spp. hybrid) is the most important sugar crop. However, its genetic improvement is severely constrained by genotype-dependent regeneration recalcitrance and lengthy tissue culture cycles. Here, we established a simple, efficient, and genotype-flexible in planta transformation system using two-leaf-stage plantlets. By systematic optimization of three key parameters: *Agrobacterium* cell density (OD_600_ = 0.5), dark incubation duration (2 weeks), and infection frequency (two rounds), we achieved a maximum transformation efficiency of 51.2%. The protocol was successfully applied to eight diverse sugarcane varieties, with transformation efficiencies ranging from 33.3% to 51.2%, demonstrating broad genotype applicability. Using this optimized system, we introduced *Sh4CL13* into sugarcane. Transgenic lines exhibited a 3.25-fold increase in *Sh4CL13* transcript levels and a 13.7-fold elevation in chlorogenic acid (CGA) content compared to controls. Feeding bioassays with *Mythimna separata* larvae revealed that transgenic lines significantly prolonged larval developmental duration, reduced pupal weight, and decreased adult emergence rates. This in planta transformation system bypasses tissue culture, offers a practical platform for functional genomics and molecular breeding in sugarcane and potentially other monocot crops.

## 1. Introduction

Sugarcane (*Saccharum* spp. hybrid) is the world’s most important sugar crop and a major feedstock for bioethanol production, contributing approximately 80% of global sugar and 40% of bioethanol [1]. However, sugarcane production is constantly threatened by various biotic and abiotic stresses, which have become critical constraints limiting yield and sugar accumulation [2]. Meanwhile, increasing population pressure and shrinking arable land have forced sugarcane cultivation to expand into marginal soils, further exacerbating the negative impacts of environmental stresses [3]. Although conventional cross-breeding has played an important role in variety improvement, the highly complex genome of sugarcane (aneuploid, polyploid, and interspecific hybrid), long breeding cycles, and difficulties in pyramiding desirable traits have made it increasingly challenging to meet the urgent demand for stress-tolerant cultivars through traditional approaches alone [4]. Consequently, genetic engineering to introduce stress-related genes into elite sugarcane varieties represents a strategic path for ensuring sustainable sugarcane production [5].

A robust and efficient genetic transformation system is the cornerstone of sugarcane molecular breeding. Currently, the mainstream techniques for sugarcane transformation remain biolistics and *Agrobacterium*-mediated transformation of callus cultures [6]. Biolistic transformation delivers exogenous DNA by high-velocity microprojectile bombardment, which is not constrained by host species but requires expensive equipment, complex operation, and often results in multi-copy random integration that can cause gene silencing and genetic instability [7]. The *Agrobacterium*-mediated transformation, which offers advantages such as low-copy-number integration and better genetic stability, has gradually become the method of choice for functional gene studies in sugarcane. Researchers have continuously improved parameters including bacterial concentration, co-cultivation conditions, and explant types to enhance transformation efficiency. However, this approach relies heavily on tissue culture and plant regeneration, which is not only time-consuming (often taking several months from transformation to regenerated plantlets) but also severely genotype-limited—most commercial sugarcane varieties have poor regeneration capacity and low transformation efficiency [8]. Furthermore, prolonged tissue culture frequently induces somaclonal variation, leading to unintended alterations in agronomic traits. Given the high heterozygosity and polyploidy of sugarcane, its thick cell walls, weak somatic regeneration capacity, and substantial genotypic variation in regenerability, the current transformation systems remain highly tissue-culture dependent, with long operational cycles, high technical barriers, elevated costs, and difficulty in establishing regeneration systems for many elite cultivars—thus seriously hampering functional genomics research and the large-scale application of molecular breeding in sugarcane [9]. Although in planta transformation may generate chimeric plants in the T_0_ generation, its simplicity and speed make it highly valuable for rapid functional gene screening and proof-of-concept studies. Therefore, there is an urgent need to explore a simpler, more efficient, and less genotype-dependent alternative transformation strategy.

In planta transformation, which delivers foreign genes directly to intact plants or detached organs without tissue culture or in vitro regeneration, has attracted considerable attention for its simplicity, short cycle, low cost, and capacity to circumvent somaclonal variation [10]. This approach was first established in *Arabidopsis* by Feldmann and Marks [11], and the subsequent development of the floral dip method by Clough and Bent [12] made in planta transformation a routine technique in *Arabidopsis*. The strategy has since been extended to various crops: in wheat, transgenic plants have been obtained by infecting shoot apical meristems or mature embryos [13]; in rice, Ho et al. [14] established a method using pulling out primary leaf from coleoptile and injection of *Agrobacterium* culture into the empty channel; in soybean, Zhong et al. [15] developed the GiFT (genotype-independent fast transformation) method combining short-term liquid culture with in planta selection, achieving efficient transformation across more than 50 elite varieties and demonstrating the enormous potential of in planta transformation for genotype flexibility. For sugarcane, Mayavan et al. [16] used stem setts as target explants and achieved 32.6% transformation efficiency by optimizing *Agrobacterium* strains, acetosyringone concentration, sonication, and vacuum infiltration parameters. Currently, reported transformation efficiencies for sugarcane vary considerably across different systems. Biolistic transformation ranges from 0.57% to 50% depending on genotypes and experimental conditions [17,18,19]. *Agrobacterium*-mediated callus transformation, though widely used, typically achieves 7.3–35.8% efficiency and remains severely genotype-dependent [20,21]. The sett-based in planta system reported by Mayavan et al. [16] achieved 32.6% efficiency but requires sonication and vacuum infiltration equipment. Collectively, these studies demonstrate the feasibility of in planta transformation for sugarcane genetic improvement. However, the sett-based method requires relatively high *Agrobacterium* concentrations and large amounts of transformation vectors, and involves a more complex operational procedure (needle-pricking, sonication, and vacuum infiltration). The stability and general applicability of the transformation system still need improvement, and systematic functional gene validation is lacking. Therefore, establishing a simpler, more easily accessible explant-based in planta transformation method with stable efficiency is of practical importance for accelerating sugarcane gene functional studies and stress-tolerant variety development.

Chlorogenic acid (CGA) is a phenolic compound that plays a central role in plant defense systems [22]. Extensive studies have demonstrated that CGA has significant inhibitory effects on various insect pests, particularly Lepidoptera. For example, feeding with 0.3% CGA to second-instar *Lymantria dispar* larvae significantly prolonged larval development, impaired molting, resulted in 100% mortality within 34 days, and completely blocked pupation and emergence [23]. Our previous work also demonstrated that CGA exerts a concentration-dependent lethal effect on third-instar *Mythimna separata* larvae, with 80% mortality at 80 mg·L^−1^ within 5 days and a sublethal concentration (LC_50_) of 7.15 mg·L^−1^, accompanied by prolonged larval developmental duration and reduced survival and fecundity [24]. CGA biosynthesis in plants relies on the interplay between the phenylpropanoid and shikimate pathways, representing a complex and multi-factor-regulated biological process [25]. 4-Coumarate:CoA ligase (4CL) serves as a hub enzyme for substrate activation and is a key rate-limiting step in CGA biosynthesis [26]. Xia et al. [27] pointed out that 4CL plays a decisive role in the metabolic flux toward CGA synthesis, catalyzing the conversion of p-coumaric acid and caffeic acid to p-coumaroyl-CoA and caffeoyl-CoA, respectively, which serve as core substrates for subsequent CGA biosynthesis. Our preliminary studies suggested that the expression of sugarcane *Sh4CL13* may influence CGA biosynthesis. Therefore, characterizing key regulatory genes in the sugarcane CGA biosynthetic pathway and elucidating their regulatory mechanisms will contribute to a better understanding of the molecular defense network of secondary metabolism and provide new references for functional studies of insect resistance genes in plants.

In this study, we used two-leaf-stage plantlets of sugarcane variety XTT22 as recipient material and developed an *Agrobacterium*-mediated shoot-tip wounding inoculation method for in planta transformation. We systematically analyzed three key parameters—*Agrobacterium* cell density, dark treatment duration, and infection frequency—for their effects on transformation efficiency to identify the optimal conditions. We then validated the genotype flexibility of this optimized protocol across multiple sugarcane varieties. Finally, using the optimized system, we introduced *Sh4CL13*, a key gene in CGA biosynthesis, into sugarcane and verified transgene integration, expression, and CGA accumulation, along with the resulting insect-resistance phenotype. This study aims to establish a simple and efficient in planta transformation system for sugarcane that can serve as a technical platform for rapid functional gene validation and molecular breeding, while also providing a methodological reference for in planta transformation in other monocot crops.

## 2. Results

### 2.1. Establishment of in Planta Transformation for Sugarcane Plantlets

**Figure 1:**
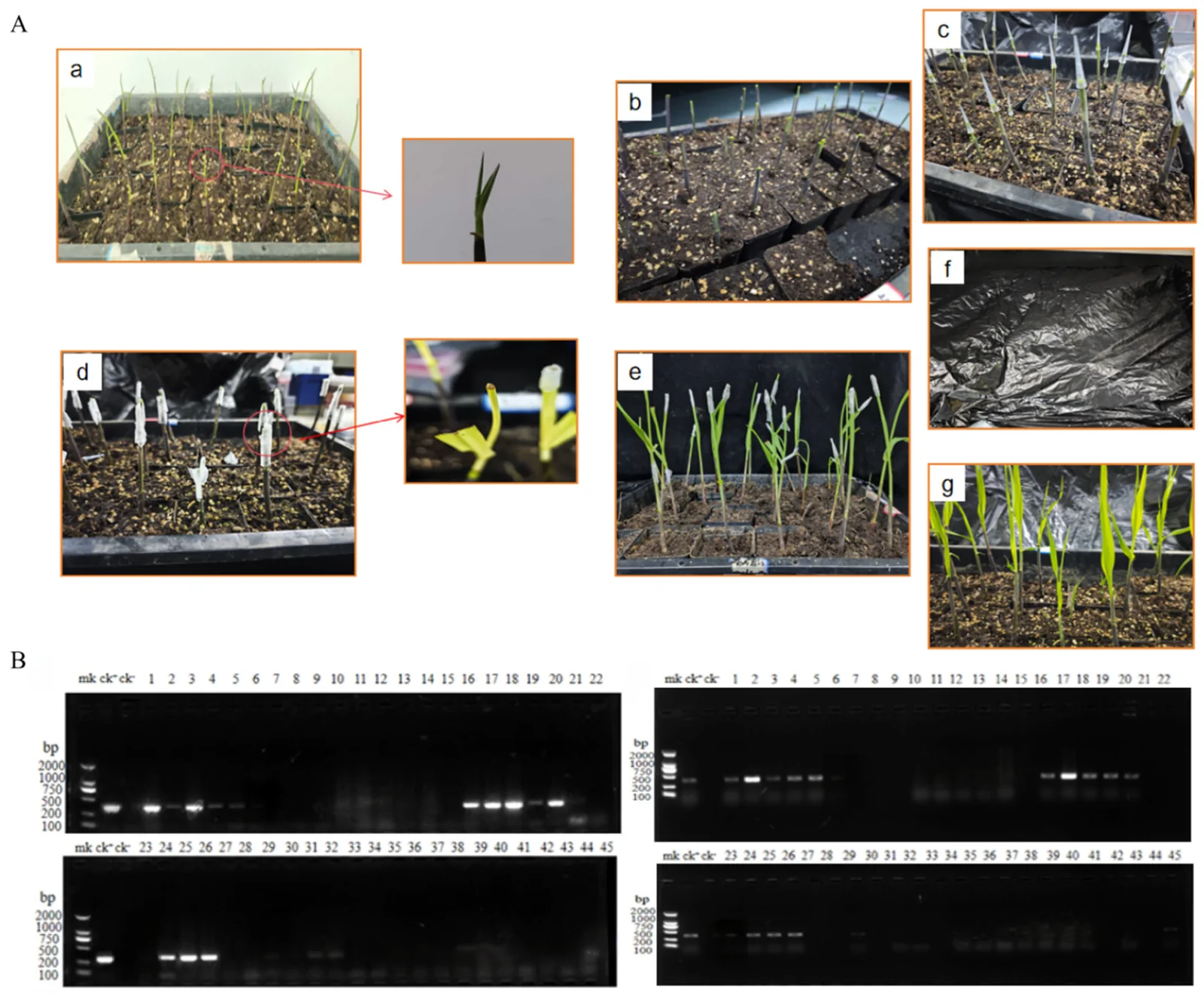
Establishment of in planta transformation for sugarcane plantlets. (**A**) Schematic diagram of the in planta transformation process for sugarcane plantlets. Note: (**a**) two-leaf-stage plantlet; (**b**) cut off the young leaves from the plantlet; (**c**) wounding and *Agrobacterium* infection; (**d**) Parafilm wrapping and dark incubation; (**e**,**f**) hygromycin selection and incubation in darkness for two weeks; (**g**) regenerated plantlets. (**B**) Detection of positive sugarcane in planta transformation by two PCR primers. The left two gels represent detection of the *RUBY* marker, and the right two gels represent detection of the plasmid marker.

Based on the established conditions (*Agrobacterium* density OD_600_ = 0.6, dark incubation for 2 weeks, and 2 rounds of infection), we conducted in planta transformation of two-leaf-stage sugarcane plantlets. After 2–3 weeks of recovery and selection, the newly regenerated shoots were visually inspected, but none exhibited the characteristic red pigmentation expected from *RUBY* expression (Figure 1A). To verify transgene integration at the molecular level, genomic DNA was extracted from the newly grown leaves and subjected to PCR analysis using two independent primer pairs. Both primer sets successfully amplified the expected fragments from the candidate lines with a positive rate of 28% (13/45), while no amplification was observed in wild-type controls (Figure 1B). These results demonstrate the establishment of a simple and efficient in planta transformation system for sugarcane.

### 2.2. Effects of Transformation Parameters on Efficiency

**Figure 2:**
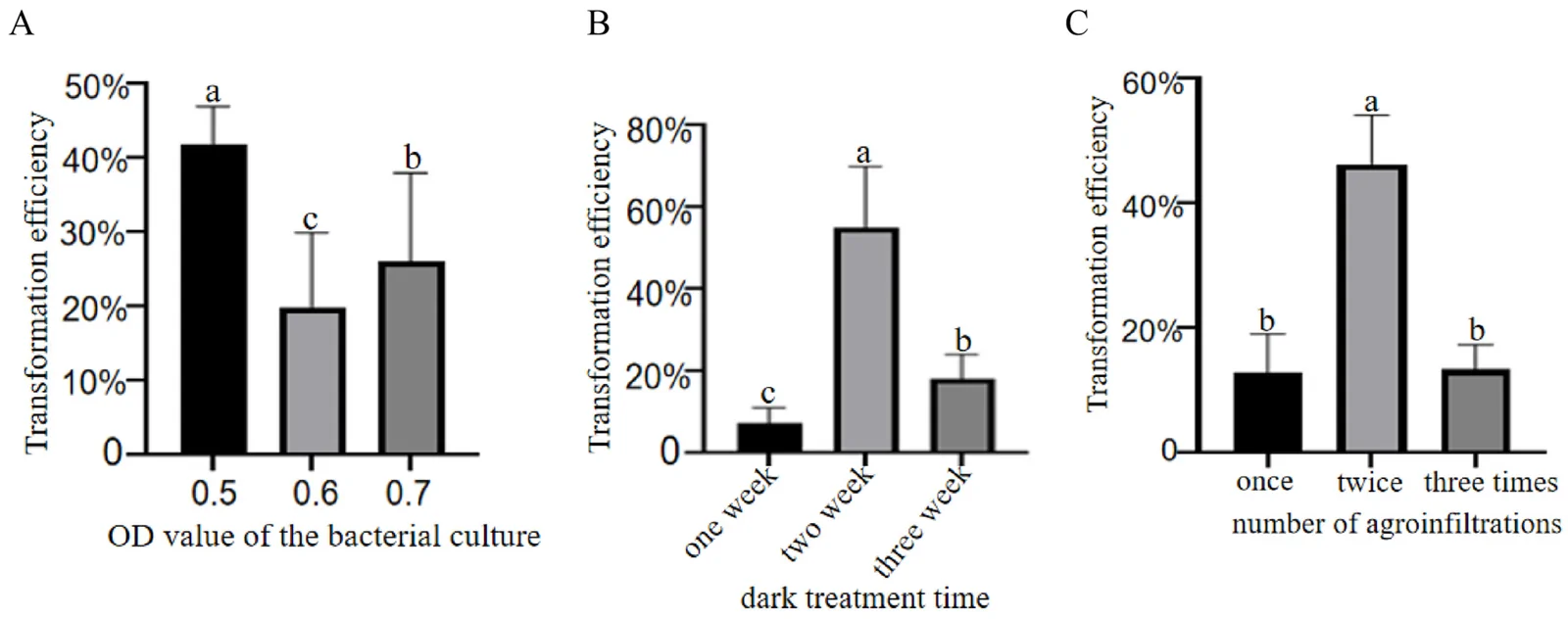
Effect of OD value (**A**), dark treatment (**B**), and number of agroinfiltrations (**C**) on the sugarcane seeding in planta transformation method. Different lowercase letters (a, b, c, etc.) above the bars indicate significant differences between treatments (Duncan’s new multiple range test, *p* < 0.05), while the same letter indicates no significant difference. Error bars represent standard error (SE).

Furthermore, to determine the optimal *Agrobacterium* cell density, we compared three OD_600_ values (0.3, 0.5, and 0.7) under conditions of 2 weeks of dark treatment and two infection rounds. As shown in Figure 2A, the highest transformation efficiency was achieved at OD_600_ = 0.5 (46.0%), which was significantly higher than that at OD_600_ = 0.3 (20.0%) and OD_600_ = 0.7 (26.0%) (*p* < 0.05). We next evaluated the effect of dark treatment duration on transformation efficiency under the optimal OD_600_ = 0.5 and two infection rounds. Dark treatment for 2 weeks gave the highest efficiency (51.2%), significantly outperforming 1 week (7.3%) and 3 weeks (17.6%) (*p* < 0.001; Figure 2B). Finally, we compared infection frequency (one, two, or three rounds) under OD_600_ = 0.5 and 2 weeks of dark treatment. Two rounds of infection yielded the highest transformation efficiency (44.6%), significantly higher than one round (13.0%) or three rounds (13.3%) (*p* < 0.001; Figure 2C).

### 2.3. In Planta Transformation Efficiency of Different Sugarcane Varieties

To assess the breadth of applicability of the optimized in planta transformation system, we transformed pCAMBIA1301-35S-*RUBY* into eight distinct sugarcane varieties using the optimal conditions (OD_600_ = 0.5, 2 weeks of dark treatment, two infection rounds). Transgenic plants were successfully obtained from all eight varieties, with transformation efficiencies ranging from 33.3% to 51.3% (Table 1), indicating the broad genotype flexibility of this method. We did not observe a significant correlation with specific agronomic traits.

### 2.4. Generation of Sh4CL13-Transgenic Sugarcane and Insect Bioassays

We cloned the complete open reading frame (ORF) of *Sh4CL13* from FN91-4621. The *Sh4CL13* consisted of 1641 bp of contiguous sequence and encoded 547 amino acids. Amino acid sequence alignment analysis revealed that the *Sh4CL13* protein contains conserved Box I (SSGTTGLPKGV) and Box II (GEICIRG) domains, as well as three additional conserved motifs: GWLHTG as the adenosine-1-phosphate binding site, FIVDRLKELKYKG FQVAPAE as the coenzyme A binding site, and SGKILRKDL as the 4CL active site (Figure S1A). Furthermore, phylogenetic tree construction showed that these 4CL proteins could be grouped into two clades. Group I corresponds to class I 4CLs from monocotyledonous plants; Group II represents 4CLs from monocotyledonous plants. The *Sh4CL13* from this study formed Group II, in which Os4CL4 was most closely related to *Sh4CL13*, followed by Zm4CL5 (Figure S1B).

**Figure 3:**
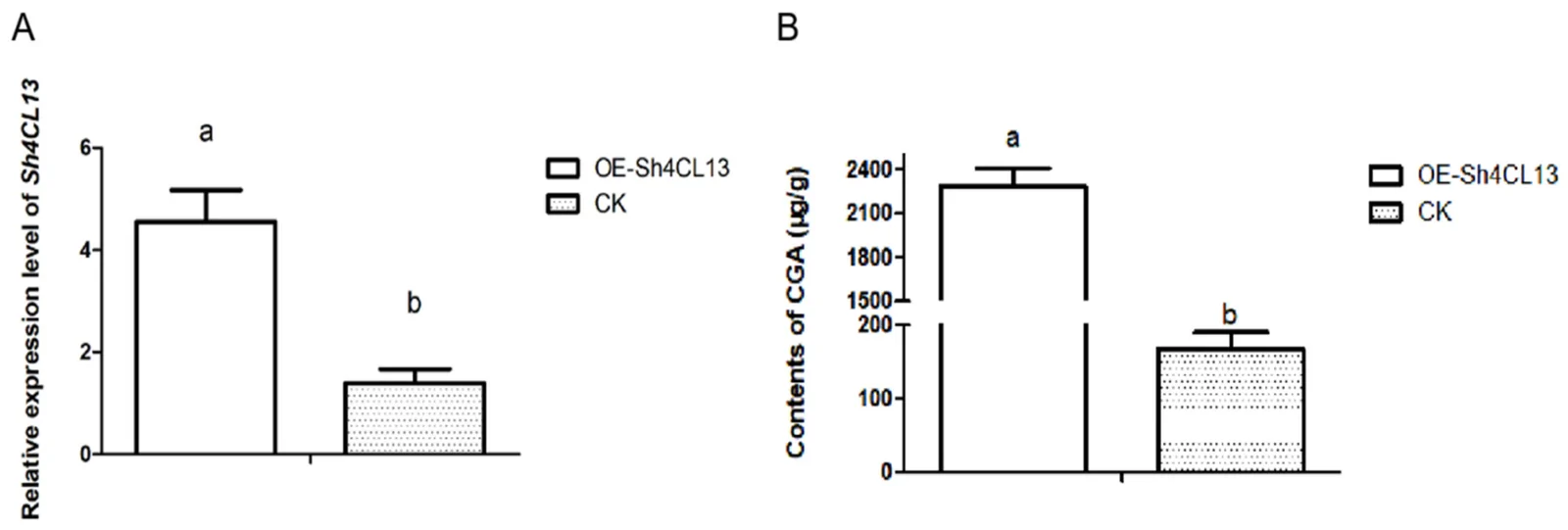
Analysis of *Sh4CL13* Gene Expression (**A**) and Chlorogenic Acid (CGA) Content (**B**) after *Sh4CL13* In planta Transformation. Different lowercase letters (a, b, c, etc.) above the bars indicate significant differences between treatments (Duncan’s new multiple range test, *p* < 0.05), while the same letter indicates no significant difference. Error bars represent standard error (SE).

Using variety FN91-4621, we generated *Sh4CL13*-overexpressing lines with the optimized protocol. Compared to EV controls, transgenic lines exhibited a 3.25-fold increase in *Sh4CL13* transcript levels (Figure 3A), accompanied by a 13.7-fold increase in CGA content (*p* < 0.05; Figure 3B).

Larvae fed on *Sh4CL13*-transgenic leaves displayed significantly impaired growth and development. Specifically, the larval developmental period was markedly prolonged (32.1 days), and pupal weight and adult emergence rates were significantly reduced compared to controls (EV and WT). However, no significant difference was observed in pupation rate (Table 2).

## 3. Discussion

In planta transformation mediated by *Agrobacterium* has gained considerable attention in plant genetic improvement and functional genomics due to its operational simplicity, lack of tissue culture requirements, and short experimental cycle [28]. However, application of this technique to monocot crops, especially sugarcane, still faces numerous challenges. *Agrobacterium*-mediated transformation efficiency is regulated by multiple factors, including bacterial density, co-cultivation conditions, and infection method, and establishing a parameter system adapted to the specific species and explant type is central to improving efficiency. In this study, using sugarcane plantlets as recipients, we systematically optimized three key parameters (*Agrobacterium* density, dark treatment duration, and infection frequency) and established an efficient in planta transformation system for sugarcane, achieving a maximum efficiency of 51.2%. This is significantly higher than most sugarcane tissue culture-based transformation systems and substantially shortens the experimental timeline by eliminating callus induction and plant regeneration steps.

The *RUBY* reporter system, based on the betalain biosynthetic pathway, requires three core conditions for visible pigmentation: complete expression of the transgene, sufficient substrate supply, and accumulation of betacyanin above the visual threshold [29]. It has been transformed into many different crops, including cotton [30], tomato [29] and maize [29]. However, in this study, PCR/RT-PCR confirmed gene integration and transcription, but no visible red phenotype was observed. Similar phenomenon were also observed in the Taigumale-sterile wheat [31] and *Pisum sativum* [32]. The most likely explanation is that betacyanin accumulation did not reach the visual threshold. We speculate that this may be due to: (1) the majority of T_0_ plants obtained through in planta transformation are chimeric—if the transformed positive cells are distributed only in the vascular bundle sheath or internal parenchyma cells rather than in epidermal or pith tissues, the trace amounts of pigment synthesized would be “diluted” by the large mass of non-transformed cells, failing to produce a macroscopic red color.

In this study, the optimal *Agrobacterium* density was OD_600_ = 0.5. This is consistent with many previous reports—for example, optimal OD_550_ ranges of 0.4–0.6 in stevia [33]—but higher than the concentrations typically used for sugarcane seed or callus systems [9]. This difference may reflect the thicker cell walls and lower accessibility of shoot apical tissues to *Agrobacterium*, requiring a moderately higher cell density to achieve sufficient infection. However, excessive bacterial density (e.g., OD_600_ = 0.7) may cause overgrowth of *Agrobacterium* at the wound site, triggering plant defense responses or tissue necrosis [34], and thus reducing transformation efficiency. Therefore, an OD_600_ of approximately 0.5 appears optimal for sugarcane seedling infection. Dark incubation is a critical step for *Agrobacterium*-mediated transformation, as dark conditions suppress plant defense responses and promote bacterial attachment and T-DNA transfer [35]. In our study, 2 weeks of dark treatment yielded the highest efficiency, while shorter or longer durations were less effective. This is consistent with the 1–3 week dark treatment commonly used in sugarcane callus transformation, but is considerably longer than the 18 h co-cultivation used in sett transformation [16] or the 2–3 days typical of standard tissue culture systems. The extended co-cultivation period may be necessary to accommodate the cell division status and wound-healing kinetics of the seedling meristem region. One week of dark treatment may be insufficient to complete efficient T-DNA transfer and integration, while 3 weeks may lead to seedling etiolation, over-healing of wounds, and reduced bacterial infection persistence. Thus, the dark treatment duration must balance T-DNA integration efficiency with plant survival. The effect of infection frequency on in planta transformation efficiency varies across species: in jujube, transformation efficiency decreases with increasing infection frequency [36], while in citrus epicotyl transformation, two infection rounds yielded higher efficiency [37]. In our study, two rounds of infection gave significantly higher efficiency than one or three rounds. We interpret this as follows: the second round of infection compensates for insufficient bacterial load in the first round, while the 3-day interval between infections minimizes sustained tissue damage and allows for tissue repair and cell division, placing more target cells in a competent state. In contrast, three rounds of infection likely exceeded the damage tolerance threshold of the plantlets, leading to concurrent declines in both survival and transformation efficiency. These results provide a reference for designing optimal infection strategies for in planta transformation in sugarcane and other monocots.

Compared with the sett-based in planta transformation method reported by Mayavan et al. [16], which employed needle-pricking, sonication, and vacuum infiltration to achieve 32.6% efficiency, our seedling shoot-tip infection method is considerably simpler, requiring neither sonication nor vacuum equipment, while still achieving higher PCR-positive efficiency (51.2%). However, it should be noted that our efficiency was based on PCR detection of the *RUBY* marker; some PCR-positive plants may represent transient expression or chimeric events. Future studies with Southern blot hybridization and progeny segregation analysis will be necessary to confirm stable transformation events.

To demonstrate the practical utility of the optimized system, we introduced *Sh4CL13*, a gene encoding 4-coumarate:CoA ligase, into sugarcane. Transgenic plants showed significantly elevated *Sh4CL13* transcript levels and CGA content, confirming that the system can effectively achieve stable transgene integration and expression. Feeding assays further showed that *Sh4CL13*-overexpressing lines significantly impaired *M. separata* development, with reduced pupal weight and adult emergence rates, and a significant negative correlation between CGA content and insect performance. In poplar, silencing *Ptr4CL3/7* reduced CGA content and increased herbivory [38], consistent with our finding that 4CL positively regulates CGA accumulation; and the induced synthesis of chlorogenic acid in sweet potato leaves enhances resistance to sweet potato weevil, suggesting that *4CL* promotes chlorogenic acid accumulation and thereby improves the defense capacity of sweet potato [39]. However, CGA biosynthesis is a complex metabolic network with multiple potential rate-limiting steps (e.g., HQT and C3H). Whether CGA content can be further linearly enhanced with increased *Sh4CL13* expression warrants further investigation. Nevertheless, our work not only enriches the functional understanding of 4CL genes but also provides a valuable gene resource and a feasible technical pathway for insect-resistant molecular breeding in sugarcane and other crops.

The in planta transformation system developed in this study offers several notable advantages: (1) it eliminates tissue culture, thereby avoiding somaclonal variation; (2) it is operationally simple, requiring no specialized equipment and amenable to wide adoption; (3) it is rapid, with the entire process from infection to molecular detection taking 4–6 weeks, enabling fast generation of transgenic materials for functional gene studies, promoter analysis, and rapid germplasm improvement; and (4) it is applicable across multiple varieties. As genome editing technologies (e.g., CRISPR/Cas9) continue to advance, this in planta system also holds promise for application in sugarcane genome editing for precision breeding. Nonetheless, there remains room for further improvement in transformation efficiency. Future optimization could explore alternative *Agrobacterium* strains (e.g., EHA105, AGL1), incorporation of nanomaterials, or vacuum infiltration assistance to further enhance efficiency and stability. Long-term follow-up studies of genetic stability and segregation patterns of the transgenes will also be important.

## 4. Materials and Methods

### 4.1. Plant Materials

Sugarcane varieties used in this study—XTT22, GT58, YZ15505, LC1539, FN91-4621, YZ14-1027, DZ03-83, and YC71-370—were maintained at the sugarcane planting base of the National Sugarcane Engineering Technology Research Center, Fujian Agriculture and Forestry University. Stem segments containing axillary buds from mature sugarcane stalks were planted in soil, and plantlets were grown under greenhouse conditions (25 ± 1 °C, 75% relative humidity, 14 h light/10 h dark photoperiod). In preliminary experiments, we compared germinating buds, two-leaf-stage, and three-leaf-stage seedlings, and found that two-leaf-stage seedlings exhibited the highest meristem activity and lowest wound necrosis; thus, two-leaf-stage plantlets were used for *Agrobacterium*-mediated transformation.

### 4.2. Insect Rearing

The oriental armyworm (*Mythimna separata*) eggs were purchased from Jiyuan Baiyun Industrial Co., Ltd. (Jiyuan, China) and maintained in a controlled growth chamber at the National Sugarcane Engineering Technology Research Center, Fujian Agriculture and Forestry University. Larvae were reared on an artificial diet according to Lin [24] under conditions of 25 ± 2 °C, 60–80% relative humidity, 10 h light/14 h dark photoperiod, and 18,000 lx light intensity.

### 4.3. Agrobacterium Strains and Binary Vectors

**Figure 4:**
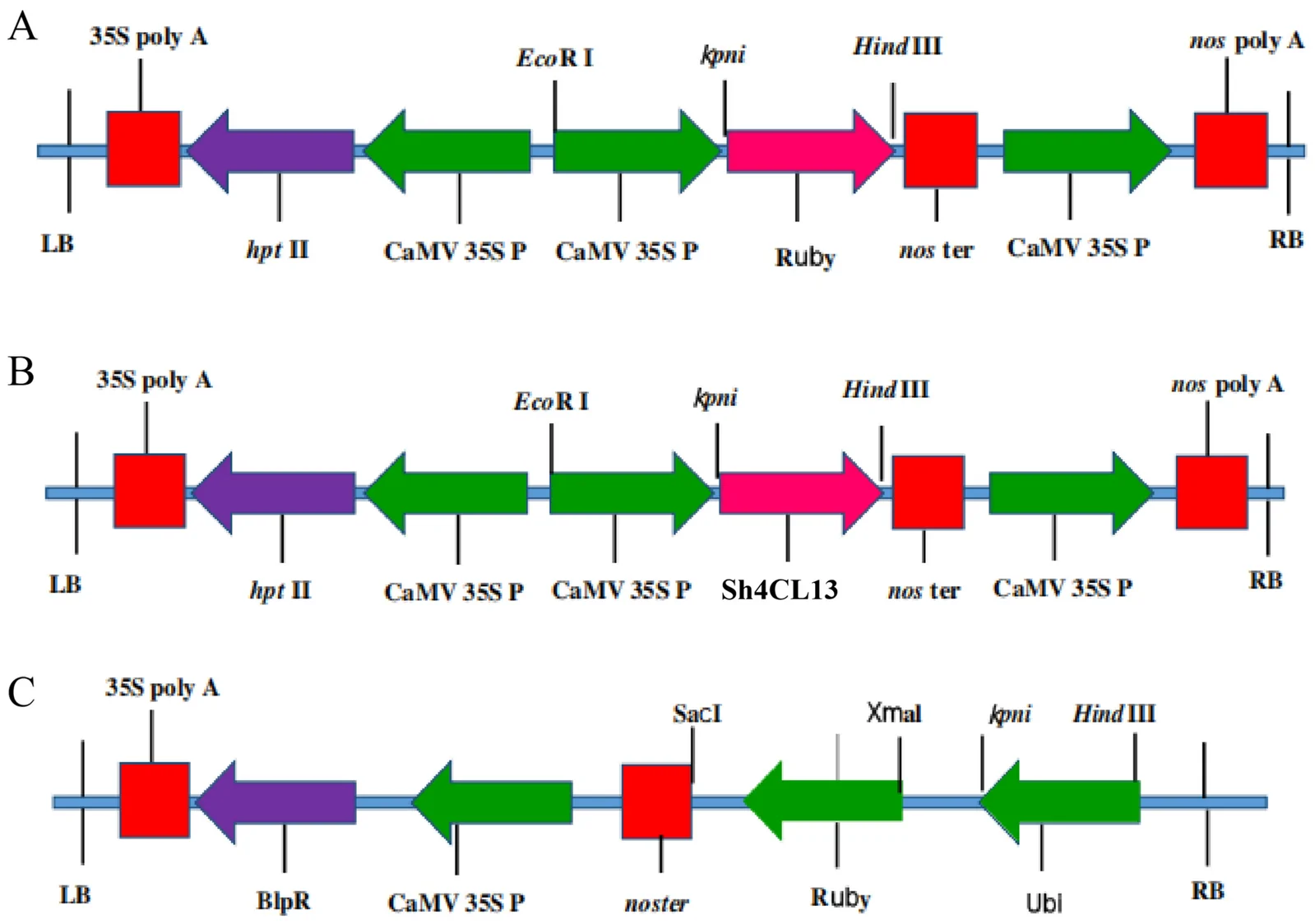
Schematic representation of the binary vector pCAMBIA1301 used in planta sugarcane setts transformation. The T-DNA region of pCAMBIA 1301 shows the assembly of the hygromycin expression cassette (CaMV 35S P: hpt II: 35S poly A) and the *RUBY* gene expression cassette (CaMV 35S P:*RUBY*: nos poly A). CaMV 35S P Cauliflower mosaic virus 35S promoter, hpt II hygromycin phosphotransferase II, 35S poly A Cauliflower mosaic virus 35S poly A terminator, nos ter nopaline synthase terminator, *RUBY* gene, nos poly A nopaline synthase poly A Terminator, UBI, Zea mays ubiquitin-1 promoter. (**A**) represents pCAMBIA1301-35S-*RUBY* plasmid; (**B**) represents pCAMBIA1301-35S-*Sh4CL13* plasmid; (**C**) represents pCAMBIA1301-UBI-*RUBY* plasmid.

*Agrobacterium* tumefaciens strain GV3101 (purchased from Weidi Biotechnology Co., Shanghai, China) was used for all transformations. Two binary vectors were constructed based on the pCAMBIA1301 backbone. pCAMBIA1301-35S-*RUBY* contained the visual reporter gene *RUBY* driven by the CaMV 35S promoter, with KpnI/HindIII cloning sites, and was used for transformation system optimization (Figure 4A). pCAMBIA1301-Ubi-*RUBY* contained the *RUBY* gene driven by the maize Ubiquitin-1 promoter, with the same restriction sites (Figure 4C). pCAMBIA1301-35S-*Sh4CL13* carried the sugarcane *Sh4CL13* coding sequence under the control of the CaMV 35S promoter (Figure 4B).

### 4.4. Cloning, Sequence and Vector Construction Analysis of Sh4CL13

Total RNA was extracted from XTT22 leaves using MagZol™ reagent (Magen, Shanghai, China), and cDNA was synthesized using HiScript^®^ II Q RT SuperMix for qPCR (Vazyme, Nanjing, China). The full-length coding sequence of *Sh4CL13* (gene ID: Sh_Ss07I0288197) was amplified from XTT22 cDNA using Ex-Taq polymerase (TaKaRa, Kyoto, Japan) with gene-specific primers (*Sh4CL13*-F and *Sh4CL13*-R, Table 1). The amplified sequences were assembled and aligned with DNAMAN (DNAMAN 5.2.2, Lynnon BioSoft). The mature *Sh4CL13* protein sequences and other plant species were aligned using ClustalX 1.83, and a phylogenetic tree was constructed in MEGA 6.0 using the neighbor-joining method with 1000 bootstrap resampling. A BstXI site was introduced at the 5′ end and an NcoI site at the 3′ end of the primers. The amplicon was double-digested and ligated into the corresponding sites of pCAMBIA1301 to generate pCAMBIA1301-35S-*Sh4CL13*. The primers used were listed in Table S1.

### 4.5. Agrobacterium Inoculum Preparation, Infection, and Transgenic Plant Recovery

GV3101 strains harboring the respective constructs were streaked onto YEP solid medium containing 50 mg/L kanamycin and 50 mg/L rifampicin and incubated at 28 °C for 2 days. A single colony was inoculated into 5 mL of YEP liquid medium with the same antibiotics and grown overnight at 28 °C with shaking at 200 rpm. A 500-μL aliquot of the pre-culture was transferred to 30 mL of fresh YEP medium and grown to logarithmic phase. The culture was centrifuged at 4000 rpm for 10 min, and the pellet was resuspended in infiltration medium (0.2132 g MES, 1 mL 1 M MgCl_2_, 100 μL acetosyringone in 100 mL, pH 5.4–5.8) to OD_600_ of 0.3, 0.5, or 0.7. Silwet L-77 was added to a final concentration of 0.02% (2 μL per 10 mL), and the suspension was incubated at 28 °C for 30 min for virulence induction. The bacterial suspension was confirmed by PCR using *RUBY*-specific primers (*Ruby*-1-F, *Ruby*-1-R; Table 1) before infection.

**Figure 5:**
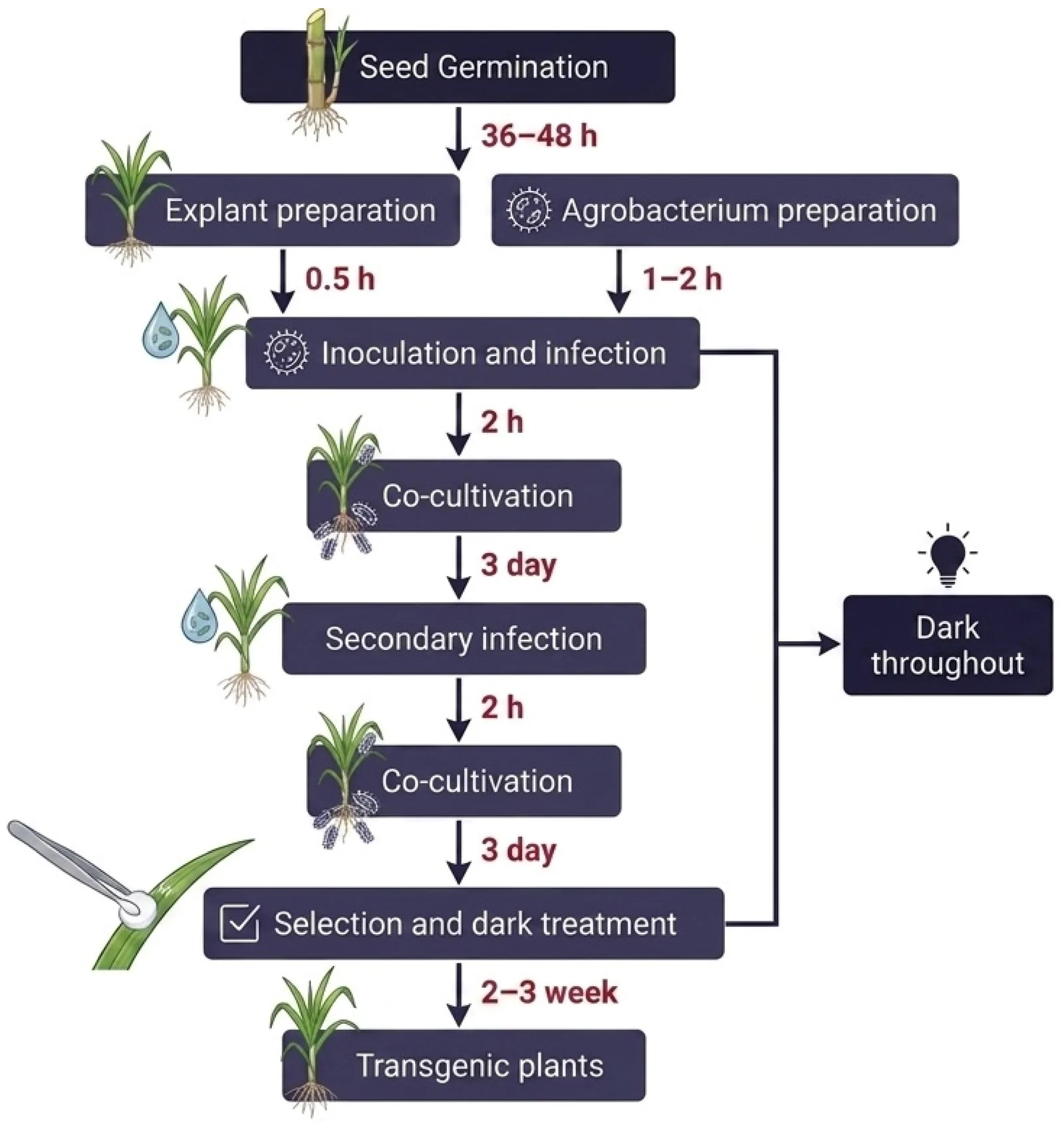
Flowchart and timeline of the sugarcane plantlets in the in planta transformation method.

Two-leaf-stage sugarcane plantlets were used for transformation [40]. Approximately 0.5 cm of the apical meristem region was excised with a sterile scalpel. A 10-μL pipette tip containing the prepared *Agrobacterium* suspension was inverted and applied directly to the wound site for 45 min. Following inoculation, the wound was wrapped with Parafilm^®^ M (Amcor Flexibles North America, Neenah, WI, USA), and the plantlets were kept in darkness for co-cultivation. A second infection was performed 3 days later using the same procedure. Three days after the second infection, the wound site was treated 2–3 times with a solution containing 50 mg/L hygromycin for selection, and again wrapped with Parafilm. The plantlets were covered with black shading cloth for dark treatment of 1, 2, or 3 weeks. After the dark treatment, plantlets were returned to normal light conditions. Each treatment consisted of three replicates with 50 plantlets per replicate. Non-transformed wild-type plantlets served as negative controls. After new leaves emerged, the number of surviving plants was recorded, and transformation efficiency was calculated (Figure 5).

Note: Two-leaf-stage plantlets were wounded at the apical meristem and inoculated with *Agrobacterium* suspension (OD_600_ = 0.5) for 45 min, followed by Parafilm wrapping and dark co-cultivation. A second infection was performed 3 days later. After the second infection, plantlets were treated with hygromycin (50 mg/L) for selection and subjected to dark incubation for 2 weeks (optimized condition), after which they were returned to normal light conditions for regeneration. Newly emerged leaves were collected for molecular detection. Arrows indicate the timeline of key steps (days/weeks post-initial infection): WT, wild-type control.

### 4.6. Molecular Detection of Transgenic Sugarcane Plants

Genomic DNA was extracted from 0.2 g of leaf tissue using a modified CTAB method. PCR was performed using primers specific to the *RUBY* marker gene (*Ruby*-1-F, *Ruby*-1-R) and vector backbone (1301-1-F, 1301-1-R; Table 2) to confirm transgene integration. The PCR conditions were: initial denaturation at 94 °C for 8 min; 35 cycles of 94 °C for 30 s, 59 °C for 30 s, and 72 °C for 1 min; and a final extension at 72 °C for 10 min. PCR products were resolved on 1.0% (*w*/*v*) agarose gels. Plasmid DNA and DNA from non-transformed plants served as positive and negative controls, respectively.

### 4.7. Generation of Sh4CL13-Transgenic Sugarcane and CGA Quantification

Using the optimized transformation protocol, pCAMBIA1301-35S-*Sh4CL13* was introduced into sugarcane variety FN91-4621. Transgenic plants, verified by PCR with vector-specific primers (1301-1-F, 1301-1-R), were used for further analysis. Total RNA was extracted using MagZol™ reagent (Magen Biotechnology Co., Ltd., Guangzhou, China), and cDNA was synthesized as described above. RT-qPCR was performed with gene-specific primers (qPCR-*Sh4CL13*-F, qPCR-*Sh4CL13*-R; Table 2) on a QuantStudio™ 6 system (Thermo Fisher Scientific, Waltham, MA, USA) using the SYBR^®^ Green method (Thermo Fisher Scientific, Waltham, MA, USA). Three biological replicates, each consisting of three plants, were analyzed.

For CGA extraction, 0.1 g of leaf tissue was rinsed with distilled water, blotted dry, cut into small pieces, and frozen in liquid nitrogen. The sample was ground in a 2.0 mL tube using a tissue homogenizer, and 500 μL of 60% ethanol was added. The mixture was incubated at 25 °C for 12 h, then centrifuged at 8000 rpm for 10 min. The supernatant (50 μL) was used as the test solution. A CGA standard curve (2.5, 5.0, 7.5, 10.0, 12.5, and 15.0 μg/mL) was prepared in 60% ethanol, and absorbance was measured at 329 nm. CGA content was calculated as described previously. Three technical replicates were performed for each biological replicate.

### 4.8. Insect Bioassay with Transgenic Sugarcane

Leaves from pCAMBIA1301-empty vector-transgenic plants (EV) and wild-type (WT) served as controls, and leaves from *Sh4CL13*-overexpressing lines were used as treatment groups. Neonatal first-instar M. separata larvae were used for life-history assays according to Lin’s work [24]. For each treatment, 15 larvae were placed individually in sterile culture cups (3 cm diameter × 2 cm height) with fresh leaf material. Three replicates were performed per treatment. Larvae were maintained in a growth chamber under the conditions described in Section 2.2. Observations were made daily; larval survival and feeding status were recorded, and fresh leaves were supplied as needed. Upon pupation, pupae were individually transferred to new cups, and pupation time and pupal weight were recorded. Adult emergence rates were scored for each treatment.

### 4.9. Statistical Analysis

Statistical analyses were performed using SPSS 22.0. One-way analysis of variance (ANOVA) was used to compare treatment means, and significant differences among treatments were determined using Duncan’s multiple range test at *p* < 0.05. Data were visualized using GraphPad Prism 8.0.

## 5. Conclusions

In this study, we established a simple, efficient, and genotype-flexible *Agrobacterium*-mediated in planta transformation system for sugarcane using two-leaf-stage plantlets as recipient explants. Using this optimized system, we demonstrated that *Sh4CL13* plays a positive role in CGA-mediated insect defense. Collectively, this work not only provides a practical and efficient technical platform for rapid functional gene validation in sugarcane but also offers a valuable methodological reference for facilitating functional genomics and molecular breeding efforts in this important crop.

## Figures and Tables

**Table 1:** Influence of genotype on in planta sugarcane transformation.

Sugarcane Variety	No. of Sugarcane Plantlets Infected	No. of Replications	Mean No. of Plantlets Sprouted	Mean No. of Surviving Plants	Positive Transformation Efficiency (%)
XTT22	50	3	43 ± 7 ^g^	39 ± 6 ^f^	51.2 ± 6.2 ^g^
GT58	50	3	38 ± 3 ^d^	33 ± 7 ^c^	36.3 ± 5.1 ^c^
YZ15505	50	3	36 ± 8 ^c^	33 ± 2 ^c^	33.3 ± 4.6 ^a^
LC1539	50	3	38 ± 7 ^d^	34 ± 5 ^d^	41.2 ± 5.3 ^e^
FN91-4621	50	3	33 ± 3 ^a^	29 ± 3 ^a^	37.9 ± 4.4 ^b^
YZ14-1027	50	3	41 ± 10 ^f^	40 ± 6 ^g^	40.0 ± 8.3 ^d^
DZ03-83	50	3	39 ± 6 ^e^	36 ± 7 ^e^	36.1 ± 4.2 ^b^
YC71-370	50	3	34 ± 7 ^b^	31 ± 5 ^b^	45.2 ± 6.3 ^f^

Note: Positive Transformation efficiency = (No. Of Positive seedling/Mean no. of surviving plants) × 100%. Different lowercase letters in the same column indicate significant differences at the 0.05 level (Duncan’s new multiple range test). Data are means (SD) or means ± SE.

**Table 2:** Effects of feeding on OE-*Sh4CL13* sugarcane Leaves on the Growth, development, and Fecundity of *M. seperata*.

Growth, Development and Fecundity Parameters	OE-*Sh4CL13*	EV	WT
Larval developmental duration (d)	32.1 ± 2.6 ^a^	29.3 ± 1.2 ^b^	28.6 ± 0.5 ^b^
Pupae weight (g)	0.220 ± 0.018 ^a^ 0.13 ^b^	0.246 ± 0.021 ^a^	0.258 ± 0.014 ^a^
Pupation rate (%)	88.3 ± 5.2 ^a^	86.4 ± 8.83 ^a^	88.4 ± 4.83 ^a^
Emergence rate (%)	68.2 ± 4.0 ^a^	79.3 ± 6.6 ^b^	82.3 ± 9.6 ^b^

Note: Different lowercase letters in the same column indicate significant differences at the 0.05 level (Duncan’s new multiple range test). Data are means (SD) or means ± SE.

## Data Availability

The authors confirm that the data supporting the findings of this study are available within the article and its Supplementary Materials.

## Supplementary Materials

The following supporting information can be downloaded at: https://www.mdpi.com/article/10.3390/plants15162489/s1. Figure S1: Sequence analysis of Sh4CL13; Table S1:Primers used in this study.
