# Supplementary material for: Development of an Optimized in Planta Transformation System in Sugarcane and Its Application on Sh4CL13 in Chlorogenic Acid Biosynthesis
Source: Plants (Basel). 2026 Aug 17;15(16):2489. doi: 10.3390/plants15162489 (PMC13516384; doi:10.3390/plants15162489)
Supplement: Supplementary file 1 [file plants-15-02489-s001.zip › plants-4472486-supplementary.pdf]

Table S1 Primers used in this study

Primer purpose	Target gene	Forward sequence	Reverse sequence
Gene cloning	Sh4CL13	ATGGTGTCGCCGACGGAGCC	TCAACAAGATGATCCGTTGG
	Ruby	ATGGATCATGCGACC	TCACTGGAGGCTTG
Plasmid Construction	Sh4CL13-1301	ACGAATTCGAGCTCGATGGTGTCGCCGACGGAGCC	GGCCAGTGCCAAGCTTCACTGGAGGCTTG
	Ruby-1301	ACGAATTCGAGCTCGATGGATCATGCGACC	GGCCAGTGCCAAGCTTCACTGGAGGCTTG
	UBI-Ruby	CTAGAGGATCCCCGAGATGGATCATGCGACC	GATCGGGGAAATTCGTCACTGGAGGCTTGGCTCAAGT
Positive plant detection	Ruby-1	TTCGCCAACCTCGCCA	TGTGGGGAGAGCAGATGCA
	1301-1	ATTTCTTTGCCCTCGGAC	GTATATGCTCCGCATTGG
Gene expression detection	qPCR-Sh4CL13	GACGGGCGAGACGCTCACG	GAGGCGAGGTCGGAGAACG

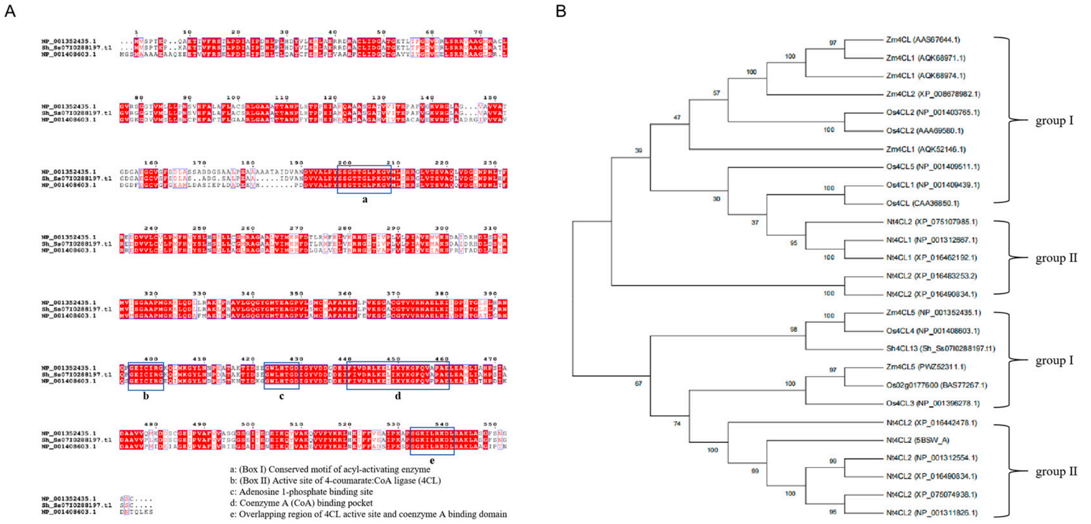

Figure S1 Sequence analysis of Sh4CL13. A. Alignment of three plant 4CLs (NP_001352435.1: Zm4CL5; Sh_Ss07I0288197.t1: Sh4CL13; NP_001408603.1: Os4CL4). B.Phylogeneic tree of Sh4CL13 and its homologues from other plants species.
